# Upregulation of MAPK pathway is associated with survival in castrate-resistant prostate cancer

**DOI:** 10.1038/bjc.2011.163

**Published:** 2011-05-10

**Authors:** R Mukherjee, D H McGuinness, P McCall, M A Underwood, M Seywright, C Orange, J Edwards

**Affiliations:** 1 College of Medical, Veterinary and Life Sciences, Institute of Cancer, McGregor Building, Glasgow Western Infirmary, Glasgow G11 6NT, UK; 2 Department of Urology, Glasgow Royal Infirmary, Glasgow G31 2ER, UK; 3 Department of Pathology, McGregor Building, Western Infirmary, Glasgow G11 6NT, UK

**Keywords:** MAP kinase pathway, castrate-resistant prostate cancer, prostate cancer, Her2, AP-1, Raf

## Abstract

**Background:**

Recent evidence has implicated the MAP kinase (MAPK) pathway with the development of castrate-resistant prostate cancer (CRPC). We have previously reported gene amplification of critical members of this pathway with the development of castrate-resistant disease. In addition, we have shown that rising Raf-1 expression, with the development of CRPC, influences time to biochemical relapse. We therefore sought to further analyse the role of both Raf-1 and its downstream target MAPK in the molecular pathogenesis of CRPC.

**Methods:**

Protein expression of Raf-1 and MAPK, including their activation status, was analysed using immunohistochemistry in a database of 65 paired tumour specimens obtained before and after the development of CRPC and correlated with other members of the pathway.

**Results:**

Patients whose nuclear expression of MAPK rose with the development of CRPC had a significantly shorter median time to death following biochemical relapse (1.40 *vs* 3.00 years, *P*=0.0255) as well as reduced disease-specific survival when compared with those whose expression fell or remained unchanged (1.16 *vs* 2.62 years, *P*=0.0005). Significant correlations were observed between protein expression of Raf-1 and MAPK with the type 1 receptor tyrosine kinases, Her2 and epidermal growth factor receptor, as well as the transcription factor AP-1 in CRPC tumours.

**Conclusion:**

We conclude that the Her2/Raf-1/MAPK/AP-1 axis may promote the development of CRPC, leading to early relapse, and reduced disease-specific survival. In addition, members of the pathway may act as novel therapeutic and/or diagnostic targets for prostate cancer.

Prostate cancer is one of the highest diagnosed forms of cancer in the western world (CancerResearchUK, 2010) and is the second most common cause of male cancer-related deaths (Debes and Tindall, 2004). If diagnosed early, the disease tends to be localised and organ specific, in these cases radical surgery and/or radiotherapy can be highly successful. However, many patients present with an advanced form, in these cases the primary form of treatment in the UK is based on androgen-ablation therapy, which was first established 50–60 years ago (Linja *et al*, 2001). This form of treatment prevents the activation of androgen-regulated genes by reducing the level of stimulation for the androgen receptor (AR), established by Huggins and Hodges (2002) this form of treatment has remained virtually unchanged for 60 years. This has been shown to effectively reduce tumour growth in 70–80% of men, and these are also known as hormone-naïve prostate cancer (HNPC). However, it is commonly accepted that all patients will eventually relapse with CRPC, for which there are very limited treatment options. The development of CRPC ultimately leads to disease progression, which is the main contributing factor for the morbidity and mortality associated with prostate cancer (Dorkin and Neal, 1997; Sciarra *et al*, 1999). Chemical control of androgens is effective in localised disease; however, for high-grade, metastatic disease, the best treatment option is surgical castration (Wirth *et al*, 2004).

Early detection of prostate cancer is hindered by an inability to distinguish between histological presence of cancerous changes that may never progress to potentially fatal metastatic disease (Culig *et al*, 2002). This indicates a great need to understand the underlying mechanisms of prostate cancer to discover new forms of detection and treatment. To address these issues, greater understanding of the mechanisms involved and development of new treatments is urgently required, as the incidence of prostate cancer is expected to increase by 20% in the next 10–25 years (Beemsterboer *et al*, 1999). Currently, levels of prostate-specific antigen (PSA) are used to determine the presence of CRPC or of progression from HNPC to CRPC (Stamey *et al*, 1987; Gao *et al*, 1997).

The Raf family of serine/threonine kinases and the MAP kinase (MAPK) pathway are heavily implicated in prostate cancer as activation of the Raf/MAPK pathway increases prostate cancer cell growth both AR-dependent and -independent under *in vitro* conditions (Yeh *et al*, 1999; Bakin *et al*, 2003). Members of the Raf family are activated by the Ras family of GTPases (Weinstein-Oppenheimer *et al*, 2000), and translocate to the cell membrane upon activation whereupon it activates MEK (MAPKK), initiating a MAPK cascade (Weinstein-Oppenheimer *et al*, 2000). Mitogen-activated protein kinase has been shown to induce Raf-1 activation, resulting in a positive feedback loop (Alessandrini *et al*, 1997; Weinstein-Oppenheimer *et al*, 2000). Mitogen-activated protein kinase has also been shown to increase the transcription of androgen-dependent genes, independently of androgens by phosphorylation of AR or its cofactors (Abreu-Martin *et al*, 1999; Ueda *et al*, 2002; Bakin *et al*, 2003; Franco *et al*, 2003). Mutations and gene expression deregulation of both Ras and Raf are associated with tumour growth and progression (Khleif *et al*, 1999; Weinstein-Oppenheimer *et al*, 2000). In addition, Raf and MEK have both been shown to be overexpressed in non-metastatic and metastatic prostate cancer cells (Weinstein-Oppenheimer *et al*, 2000; Fu *et al*, 2003). Mitogen-activated protein kinase activity has been shown to be elevated in CRPC (Abreu-Martin *et al*, 1999; Gioeli *et al*, 1999), in which activated MAPK is believed to be able to stimulate prostate cancer cell growth independently of AR by activating various transcription factors including AP-1 (a homo- or heterodimer of phosphorylated c-jun and c-fos) and c-myc (Weinstein-Oppenheimer *et al*, 2000). In fact, increased AP-1 expression has been linked to castrate resistance and decreased patient survival (Edwards *et al*, 2004). In addition, it has been shown that Raf-1 expression increases with the development of CRPC and there is a significantly shorter time to relapse (Mukherjee *et al*, 2005).

Although the Ras/Raf/MAPK pathway has been implicated in the development of CRPC through cell-line and xenograft models (Abreu-Martin *et al*, 1999; Bakin *et al*, 2003), its precise role in the development of clinical CRPC is still poorly understood. The further elucidation of its role would clearly have therapeutic implications, as *in vitro* studies suggest inhibition of the pathway in conjunction with hormone therapy or chemotherapy may provide a novel method of treatment in CRPC (Bakin *et al*, 2003; Zelivianski *et al*, 2003).

Here we evaluate and discuss the expression levels of Raf-1 and MAPK (critical members of the MAPK cascade) and their activation status in matched HNPC and CRPC tumours and correlate expression levels with type 1 tyrosine kinase receptors, as well as their downstream targets, AR and AP-1, in the same cohort of patients.

## Patients and methods

### Patients

A total of 65 subjects who were recruited and met the specific inclusion criteria of having developed CRPC, this was determined by: firstly an initial response to androgen deprivation therapy, defined as a fall in PSA levels of at least 50% following orchidectomy or LHRH agonists and anti-androgen treatment. Secondly, all subjects showed a subsequent relapse (defined as a sustained rise in PSA levels despite maximal treatment). These subjects were identified retrospectively and samples were retrieved from archived stored specimens. Formalin-fixed tumour specimens from initial diagnosis (before any therapy) and post-CRPC development were obtained for this study, providing hormone-naïve and castrate-resistant samples for each individual, respectively. A total of 65 prostate cancer patients were included in this study (130 tumours in total) (diagnosed between 1984 and 2000). All tumours had patient identification removed, and the clinical information database was anonymised. Ethical approval was obtained from the Multicentre Research Ethics Committee for Scotland (MREC/01/0/36) and Local Research and Ethical Committees. Patients were only selected for analysis if they initially responded to hormone treatment (in the form of subcapsular bilateral orchidectomy or maximum androgen blockade), but subsequently relapsed (two consecutive rises in PSA greater than 10%) and had a pre- and post-castrate-resistant tissue sample available for analysis. Hormone-naïve tissue was obtained from 18 patients by TRUS-guided biopsy and the remaining by TURP, and castrate-resistant tumours were all obtained by TURP. Information on AR, PSA (Edwards *et al*, 2003a), HER2, epidermal growth factor receptor (EGFR) 6 (Edwards *et al*, 2006) and AP-1 (Edwards *et al*, 2004) expression levels and immunohistochemistry in each of these samples had been identified previously.

### Immunohistochemistry

Five micrometer paraffin-embedded tissue sections were used for immunohistochemistry staining. Briefly, sections were de-paraffinised with xylene and rehydrated. Sections were then incubated in epitope retrieval solution (DakoCytomation, Glostrup, Denmark) for 20 min at 96°C, and cooled down to room temperature. After antigen retrieval, sections were blocked with horse serum (1.5% horse serum/TBS) for 30 min, followed by overnight incubation at 4°C with appropriate primary antibodies. Overall Raf-1 expression was assayed with mouse monoclonal antibodies (1 : 20 dilution; Santa Cruz Biotechnology Inc., Santa Cruz, CA, USA), whereas the level of inactivated Raf-1 was analysed using rabbit polyclonal antibodies (1 : 25 dilution; Cell Signaling Technology, Irvine, CA, USA). Phosphorylated Raf-1 (pRaf-1) (Ser338) was detected with a rat monoclonal antibody diluted 1 : 250 (Upstate Biotechnology, Lake Placid, NY, USA). Total p42/p44 (MAPK) as well as activated p42/p44 (phosphorylated at Thr202/204) was assessed using rabbit polyclonal antibodies (1 : 25 and 1 : 50 dilutions, respectively; Cell Signaling Technology). After primary antibody incubation, tissue sections were washed with TBS and incubated with biotinylated species-specific secondary antibodies (DakoCytomation). After subsequent washes with TBS, streptavidin/HRP complexes and DAB chromogene were used to visualise staining. Tissue sections were then counterstained with haematoxylin.

### Histoscoring

All immunostained tumour sections were quantified blindly and independently by two observers, using a weighted histoscore method at high magnification ( × 400). Specific staining in each tissue section was allocated 0 (no staining), 1 (weak intensity of staining), 2 (moderate intensity) or 3 (strong intensity). The final score (maximum 300) is calculated from the sum of: 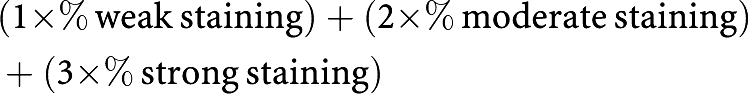


### Statistical analysis

Statistical analysis was performed using the SPSS statistical package (version 9.0). Protein expression data was not normally distributed and is shown as median and inter-quartile ranges. Wilcoxon signed rank tests were used to compare expression between HNPC and CRPC tumours. Correlations (nonlinear) between proteins were calculated using the Spearman rank test. Time to relapse and survival analysis was conducted using the Kaplan–Meier method and curves were compared with the log-rank test.

Strong correlations are defined as having a *P*<0.01 and *r*^2^⩾0.25; moderate correlations are defined as having a *P*<0.05 and *r*^2^<0.25, but ⩾0.1; and weak correlations are defined with a *P*<0.05 and *r*^2^<0.1.

## Results

### Patient cohort characteristics

The clinical data collected and recorded for each patient included age (median: 70 years; inter-quartile range: 66–74 years), PSA level at diagnosis (median: 31 ng ml^−1^; inter-quartile range: 7.8–109 ng ml^−1^), PSA level at relapse (median: 10 ng ml^−1^; inter-quartile range: 4–11 ng ml^−1^) and Gleason grade at diagnosis (median: 8, inter-quartile range: 7–9). All patients underwent biochemical relapse (median time to relapse: 2.54 years; inter-quartile range: 1.51–4.62 years). All patients included in this study were deceased (median time to death after relapse: 1.37 years; inter-quartile range: 0.82–2.69). This resulted in an overall median survival time of 4.5 years (inter-quartile range: 3.00–7.01). Patients were diagnosed with locally advanced (46 patients) or metastatic (19 patients) prostate cancer, and at the time of relapse the number of patients presenting with metastatic disease had risen (40 patients). At diagnosis, patients either underwent surgery (27 orchidectomy) or androgen deprivation therapy (59 GnRH analogue and/or anti-androgen deprivation therapy). Most patients were deceased during the course of follow-up (46 patients), although some survived beyond follow-up (19 patients).

The presence of metastatic disease at diagnosis was associated with reduced time to biochemical relapse (*P*=0.037) and a trend in overall patient survival (*P*=0.06). High Gleason scores were associated with more rapid biochemical relapse (*P*=0.002), time to death after biochemical relapse (*P*=0.02) and disease-specific survival (*P*=0.001), confirming the association of these clinical parameters with the progression of prostate cancer.

### Expression and localisation of Raf-1 and MAPK

**Figure 1:**
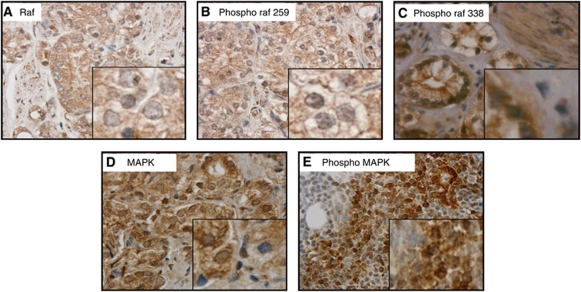
Raf and MAPK localisation in prostate cancer. Tissue sections counterstained with haematoxylin showing (magnification × 400) (**A**) total Raf; (**B**) inactive pRaf (Ser259); (**C**) active pRaf (Ser338); (**D**) total MAPK; and (**E**) activated (phospho) MAPK localisations in prostate cancer. Inset shows higher magnification images.

Raf-1 was observed in the cytoplasm and peri-membrane areas (Figure 1A), and the inactive form of Raf-1 was found only in the peri-membrane region (Figure 1B); however, the activated form of Raf-1 was observed in the cytoplasm, nucleus and the cell membrane (Figure 1C). Mitogen-activated protein kinase and phosphorylated MAPK (pMAPK) (Thr202/204) were observed in the nucleus and cytoplasm (Figure 1D).

Overall, comparison of HNPC tumours and CRPC tumours revealed no differences in total or average expression levels of either Raf-1 (and all phosphorylated forms) (Table 1) or MAPK (activated or inactivate, nuclear or cytoplasmic) (Table 2). However, analysis of matched pairs revealed a subgroup that showed significant increases in Raf-1 and MAPK levels in CRPC tumours.

**Figure 2:**
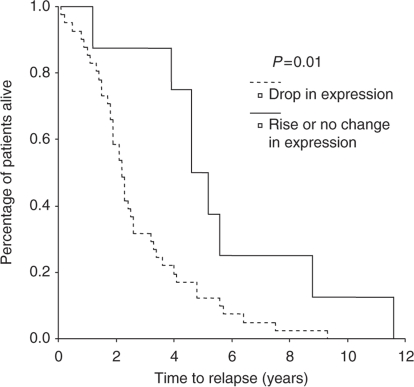
Kaplan–Meier plot for patients who showed a significant fall in pRaf (ser338) expression with the development of CRPC compared with those who did not. There was a significant difference in time to relapse in these patients (*P*=0.01).

We have previously reported that patients whose Raf-1 expression rose in this cohort with the development of CRPC had a significantly shorter time to relapse than those patients who had a fall or no change (*P*=0.0005) (Mukherjee *et al*, 2005). Consistent with this observation, patients with rising levels of nuclear pRaf (Ser338, activated Raf-1) had a significantly shorter time to biochemical relapse (2.2 years; range: 1.84–2.56 years) by 2.4 years when compared with patients who showed a fall in levels (4.6 years; range: 3.4–5.8 years) (*P*=0.01) (Figure 2).

**Figure 3:**
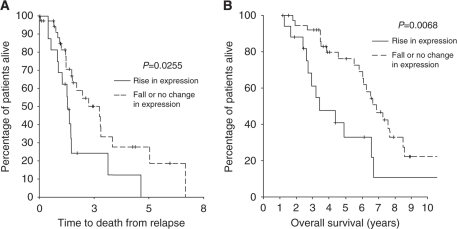
Kaplan–Meier plot for patients who showed a rise in nuclear MAPK expression with the development of CRPC compared with those who did not. There was a significant difference in (**A**) time to death from relapse in these patient subgroups (*P*=0.0255) and (**B**) disease-specific survival in these patient subgroups (*P*=0.0068).

Patients whose nuclear MAPK expression rose with the development of CRPC had a significantly shorter time to death from relapse (1.40 (1.20–1.61) years) compared with samples that showed a fall or no change in expression (3.00 (1.43–4.57) years), *P*=0.0255 (Figure 3A). This translated into a shorter disease-specific survival of 3.37 (1.58–5.16 ) years compared with 6.89 (5.70–8.08) years, *P*=0.0068 (Figure 3B). Interestingly, there was also an association seen between rising cytoplasmic pMAPK (Thr202/204) expression and reduced time to biochemical relapse (*P*=0.06).

### Relative protein expression levels in hormone-naïve prostate cancer

In HNPC tumours, Raf-1 correlated with the inactive form, pRaf (Ser259), before relapse (*P*=0.0041, *r*^2^=0.1201). Nuclear pRaf (Ser338) and cytoplasmic pRaf (Ser338) strongly correlated with each other (*P*=0.0035, *r*^2^=0.1381). No correlations between total Raf-1 and cytoplasmic pRaf (Ser338) or nuclear pRaf (Ser338) expression were evident. In addition no correlations were observed between the inactive form of pRaf (Ser259) and nuclear pRaf (Ser338) or cytoplasmic pRaf (Ser338).

Total cytoplasmic expression of MAPK correlated with total nuclear expression of MAPK (*P*=0.0126, *r*^2^=0.1009), activated MAPK (Thr202/204) (*P*=0.0152, *r*^2^=0.1044) and the cytoplasmic expression of MAPK (Thr202/204) (*P*<0.0001, *r*^2^=0.3391). Total nuclear MAPK expression correlated very strongly with the activated form of MAPK (Thr202/204) in the nucleus (*P*<0.0001, *r*^2^=0.3447) and pMAPK (Thr202/204) in the cytoplasm (*P*=0.0075, *r*^2^=0.1250). Activated cytoplasmic MAPK (Thr202/204) also very strongly associated with its activated expression (MAPK pThr202) in the nucleus (*P*<0.0001, *r*^2^=0.5210).

In addition, Raf-1 and pRaf (Ser259) both correlated with the cytoplasmic expression of MAPK (*P*<0.0001, *r*^2^=0.2979 and *P*=0.0005, *r*^2^=0.1879, respectively) and activated cytoplasmic MAPK (Thr202/204) (*P*=0.0007, *r*^2^=0.1826 and *P*=0.0069, *r*^2^=0.1214, respectively). Raf-1 also correlated with nuclear activated MAPK (Thr202/204) (*P*=0.0022, *r*^2^=0.1525). There were no correlations seen between pRaf (Ser338) and MAPK or pMAPK (Thr202/204).

### Relative protein expression levels in CRPC

In CRPC tumours, Raf-1 correlated with pRaf (Ser259) strongly (*P*=0.0003, *r*^2^=0.1778). The nuclear and cytoplasmic forms of pRaf (Ser338) also strongly correlated with each other (*P*<0.0001, *r*^2^=0.6009). Similarly to HNPC, protein expression of the inactive form of pRaf (Ser259) and the active forms of pRaf (Ser338) did not correlate in the cell cytoplasm or nucleus.

After the development of CRPC, cytoplasmic expression of total MAPK became strongly associated with the expression of total MAPK in the nucleus (*P*=<0.0001, *r*^2^=0.2564) and more weakly with its activated form, pMAPK (Thr202/204), in the nucleus (*P*=0.0194, *r*^2^=0.0864). Cytoplasmic expression of total MAPK continued to show a correlation with its activated form in the cytoplasm (MAPK pThr202/204) (*P*=0.0004, *r*^2^=0.1861). Activated cytoplasmic MAPK (Thr202/204) remained strongly associated with activated nuclear MAPK (Thr202/204) (*P*<0.0001, *r*^2^=0.5350).

Raf-1 showed similar associations with cytoplasmic and nuclear MAPK expression (*P*<0.0001, *r*^2^=0.2141 and *P*=0.0016, *r*^2^=0.1426, respectively). Cytoplasmic MAPK also correlated with pRaf (Ser259) (*P*=0.0026, *r*^2^=0.1329). Similarly to HNPC tumours, no correlations were noted between pRaf (Ser338) and MAPK in any location or form.

### Relative expression levels of Raf-1 and MAPK compared with upstream and downstream targets in hormone-naïve prostate cancer

Analysis of the upstream activators EGFR and Her2, as well as the downstream targets AR and AP-1, has previously been carried out on the same cohort of patients (Edwards *et al*, 2003a, 2004; Bartlett *et al*, 2005). We therefore correlated Raf-1 and MAPK expression with these previous results, to determine the differences in the entire pathway in the transition from HNPC to CRPC.

**Figure 4:**
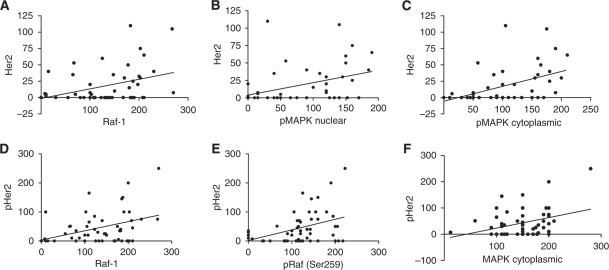
Raf-1 and MAPK show correlations with Her2 and activated Her2 (pHer2) in HNPC tumours. (**A**) Her2 correlation with Raf-1 (*P*=0.0099, *r*^2^=0.1333); (**B**) Her2 correlation with pMAPK nuclear (*P*=0.015, *r*^2^=0.13); (**C**) Her2 correlation with pMAPK cytoplasmic (*P*=0.0017, *r*^2^=0.2074); (**D**) pHer2 correlation with Raf-1 (*P*=0.0056, *r*^2^=0.1435); (**E**) pHer2 correlation with pRaf (Ser259) (*P*=0.0052, *r*^2^=0.1459); and (**F**) pHer2 correlation with MAPK cytoplasmic (*P*=0.0123, *r*^2^=0.1211).

In hormone-naïve tumours, expression of the putative growth factor receptor Her2 correlated with Raf-1 expression (*P*=0.0099, *r*^2^=0.1333) and the nuclear and cytoplasmic expression of pMAPK (Thr202/204) (*P*=0.0150, *r*^2^=0.1300 and *P*=0.0017, *r*^2^=0.2074, respectively). Once activated, the phosphorylated form of Her2 (pHer2) showed moderate correlations with Raf-1 (*P*=0.0056, *r*^2^=0.1435), pRaf (Ser259) (*P*=0.0052, *r*^2^=0.1459) and cytoplasmic MAPK expression (*P*=0.0123, *r*^2^=0.1211) (Figure 4), but not the activated forms of Raf or MAPK.

No correlations were seen with EGFR in HNPC tumours, except weak correlations with Raf-1 (*P*=0.0439, *r*^2^=0.06008) and pMAPK (Thr202/204) (nuclear) (*P*=0.0243, *r*^2^=0.0844). However, the mutant variant, EGFR vIII, did correlate strongly with cytoplasmic expression of MAPK (*P*<0.0001, *r*^2^=0.2617) and to a weaker extent with Raf-1 (*P*=0.025, *r*^2^=0.07965) and pRaf (Ser259) (*P*=0.0323, *r*^2^=0.07295).

**Figure 5:**
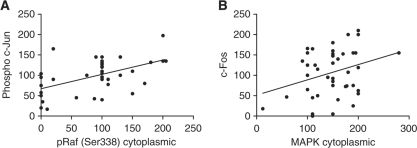
Cytoplasmic pRaf (Ser338) and cytoplasmic MAPK correlate with phospho c-Jun and c-Fos, respectively, in HNPC. (**A**) Phospho c-Jun correlation with pRaf (Ser338) cytoplasmic (*P*=0.0009, *r*^2^=0.2673) and (**B**) c-Fos correlation with MAPK cytoplasmic (*P*=0.0453, *r*^2^=0.0942).

**Figure 6:**
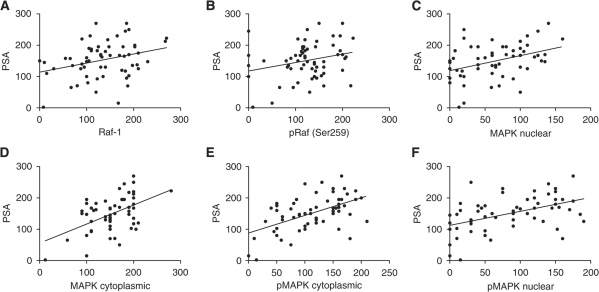
Prostate-specific antigen correlates with Raf and MAPK in HNPC tumours. (**A**) PSA correlation with Raf-1 (*P*=0.0142, *r*^2^=0.096); (**B**) PSA correlation with pRaf (Ser259) (*P*=0.043, *r*^2^=0.0655); (**C**) PSA correlation with MAPK nuclear (*P*=0.0029, *r*^2^=0.1502); (**D**) PSA correlation with MAPK cytoplasmic (*P*<0.0001, *r*^2^=0.2467); (**E**) PSA correlation with pMAPK cytoplasmic (*P*<0.0001, *r*^2^=0.2891); and (**F**) PSA correlation with pMAPK nuclear (*P*=0.0001, *r*^2^=0.2233).

Further downstream, activated pRaf (Ser338) in the cytoplasm strongly correlated with phosphorylated c-jun (*P*=0.0009, *r*^2^=0.2673) and total MAPK (cytoplasmic) correlated weakly with c-Fos (*P*=0.0453, *r*^2^=0.09418) (Figure 5). There were no correlations seen between MAPK and its activated form with c-jun, phospho c-Jun or c-Fos. In addition, PSA showed weak correlations with Raf-1 (*P*=0.0142, *r*^2^=0.09604), pRaf (Ser259) (*P*=0.043, *r*^2^=0.06547), and showed links to overall MAPK levels, MAPK (nuclear) (*P*=0.0029, *r*^2^=0.1502) and MAPK (cytoplasmic) (*P*<0.0001, *r*^2^=0.2467), with strong ties to the activated forms, pMAPK (Thr202/204) (cytoplasmic) (*P*<0.0001, *r*^2^=0.2891) and pMAPK (Thr202/204) (nuclear) (*P*=0.0001, *r*^2^=0.2233) (Figure 6), whereas AR showed no correlations with Raf, MAPK or any of their forms.

### Relative expression levels of Raf-1 and MAPK compared with upstream and downstream targets in CRPC

**Figure 7:**
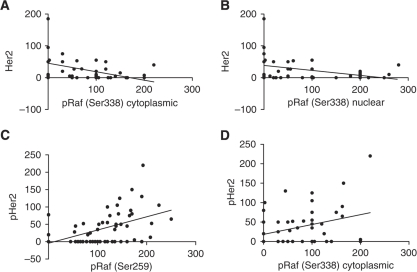
Activated Raf correlates with Her2 and activated Her2 (pHer2) in CRPC tumours. (**A**) Her2 correlation with pRaf (Ser338) cytoplasmic (*P*=0.0035, *r*^2^=0.1854); (**B**) Her2 correlation with pRaf (Ser338) nuclear (*P*=0.0068, *r*^2^=0.1581); (**C**) pHer2 correlation with pRaf (Ser259) (*P*=0.0008, *r*^2^=0.2011); and (**D**) pHer2 correlation with pRaf (Ser338) cytoplasmic (*P*=0.0363, *r*^2^=0.0938).

After the development of CRPC, Her2 no longer showed a correlation with Raf-1 nor a negative association became evident between pRaf (Ser338) both in the cytoplasm (*P*=0.0035, *r*^2^=0.1854) and nucleus (*P*=0.0068, *r*^2^=0.1581) with Her2. In addition, the activated form (pHer2) correlated with pRaf (Ser259) (*P*=0.0008, *r*^2^=0.2011). Interestingly, the activated cytoplasmic form of Raf showed a positive weak correlation with the pHer2 (*P*=0.0363, *r*^2^=0.09376) (Figure 7). Furthermore, no correlations were evident between Her2 or pHer2 with MAPK in any form. All associations between EGFR and EGFR vIII disappeared after progression to CRPC.

**Figure 8:**
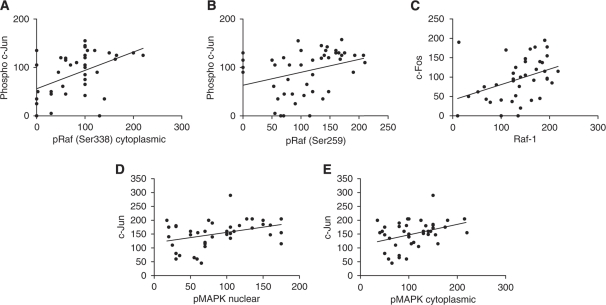
Raf and activated MAPK correlate with components of AP-1 in CRPC. (**A**) Phospho c-Jun correlation with pRaf (Ser338) cytoplasmic (*P*=0.0026, *r*^2^=0.2192); (**B**) phospho c-Jun correlation with pRaf (Ser259) (*P*=0.0278, *r*^2^=0.1077); (**C**) c-Fos correlation with Raf-1 (*P*=0.0131, *r*^2^=0.1512); (**D**) c-Jun correlation with pMAPK nuclear (*P*=0.0146, *r*^2^=0.1368); and (**E**) c-Jun correlation with pMAPK cytoplasmic (*P*=0.0178, *r*^2^=0.1295).

A moderate positive correlation was seen between pRaf (Ser259) and AR (*P*=0.0035, *r*^2^=0.1154), whereas a negative correlation is evident between nuclear MAPK and AR (*P*=0.0429, *r*^2^=0.06066). There was no correlation (positive or negative) shown between total Raf-1 expression and AR. In addition, there were no correlations noted between either Raf-1 (any form) or MAPK (any form) and PSA level. More correlations were noted with components of the downstream transcription factor AP-1. Correlations were evident between phosphorylated c-jun and cytoplasmic pRaf (Ser338) (*P*=0.0026, *r*^2^=0.2192) and pRaf (Ser259) (*P*=0.0278, *r*^2^=0.1077). There was also a correlation seen between Raf-1 and c-Fos (*P*=0.0131, *r*^2^=0.1512), and between the nuclear and cytoplasmic expression of pMAPK (Thr202/204) and c-jun (*P*=0.0146, *r*^2^=0.1368 and *P*=0.0178, *r*^2^=0.1295, respectively) (Figure 8).

## Discussion

The MAPK pathway is known to have critical roles in the control of the cell cycle, growth and prevention of apoptosis (Bradham and McClay, 2006). This pathway has been implicated in the development of CRPC and circumvention of AR involvement in prostate cancer (Edwards *et al*, 2003b). Raf is well known to be involved in a broad range of cancers (Jilaveanu *et al*, 2009) and is considered to be an oncogene that drives the MAPK pathway and proliferation to enhance tumourigenesis (Karreth *et al*, 2009). The upstream activator of Raf, Ras, has also previously been identified as being linked to tumourigenesis (Edwards and Bartlett, 2005).

The distribution of Raf-1 (Roskoski, 2010) and MAPK (Cuadrado and Nebreda, 2010) observed by immunohistochemistry is consistent with the current understanding of their cellular localisation and functions, indicating that their localisation and functions were not disrupted during tumourigenesis, their levels however showed a very broad range of fluctuations. The data presented here suggests that Raf-1 activates MAPK in the cytoplasm, which in turn translocates to the nucleus. No correlations were observed between activated Raf-1 and activated MAPK; however, this was not unexpected as Raf-1 becomes rapidly inactivated and phosphorylated at serine 259 in the cytoplasm.

Raf has been shown to have the ability to circumvent the MAPK pathway by acting directly upon retinoblastoma protein (Dhillon *et al*, 2003; Dasgupta *et al*, 2004). The very strong correlations between cytoplasmic and nuclear activated Raf levels and the weaker correlations between Raf and MAPK may indicate that a small portion of Raf activity may be attributable to MAPK-independent action. The lack of any clear links between active Raf and active MAPK in CRPC would appear to indicate that the two pathways can and do operate independently in the latter stages of prostate cancer. In addition, the tight correlations present between the various forms of MAPK after transition to CRPC is a strong indicator that this pathway has become very active in either the transition to or progression of CRPC.

There are several correlations between PSA levels and both Raf and MAPK (all forms) indicating a clear and distinct link between PSA and these pathways in HNPC. Interestingly, these correlations are completely absent in CRPC, giving further indication that these pathways have become dislocated with the general indicators associated with prostate cancer (i.e., PSA and Gleason score). This may indicate a reason for PSA being useful in the initial diagnosis of prostate cancer, but not in determining hormone relapse or overall survival (Supplementary Figure 1). Furthermore, this may indicate that markers in the Raf and MAPK pathways may be more appropriate targets for monitoring disease progression.

Interestingly, there are links between active MAPK (Thr202/204) and HER2 in HNPC, which are completely absent in CRPC; in addition, there are no links between pRaf (Ser338) and Her2 or pHer2 in HNPC, which shifts to negative correlations between pRaf (Ser338) and Her2 in CRPC. Intriguingly, there is a complete swing from activated cytoplasmic Raf (Ser338) from this negative correlation with Her2 to a positive correlation with pHer2. This may be an indicator that the Her2 signalling pathway has become active and is signalling through Raf; this idea is enhanced by the correlation of cytoplasmic activated Raf (Ser338) with phospho c-Jun. Taken together, these data suggest that Her2 is signalling through Raf and ultimately through c-Jun (AP-1), as we have suggested previously (Edwards *et al*, 2004), to circumvent AR activity after the tumour has become insensitive to androgens, as AP-1 has been shown to act independently of AR to activate some of the AR-associated genes (Sato *et al*, 1997). Furthermore, the links between activated MAPK and c-Jun, but a lack of any links with Her2 or EGFR, is further evidence that MAPK and Raf are acting independently of each other to achieve the same end goal. Both MAPK and PKC have been shown to phosphorylate c-Jun directly to stabilise it (Sato *et al*, 1997; Sadar *et al*, 1999; Bonaccorsi *et al*, 2003); however, the lack of correlation between MAPK and phospho c-Jun, in addition to our previous findings that phopho c-Jun is upregulated in 61% of cases (Edwards *et al*, 2004), suggests that MAPK is not acting through this mechanism. In fact, the evidence indicates that MAPK may have a role in c-Jun activation of AR, by promotion of dimerisation (Wise *et al*, 1998), in the absence of hormones. This provides a mechanism for the activation of androgen-activated genes without the need for the androgen signal, thus making the tumour insensitive to hormone withdrawal. The links between Raf and the components of AP-1 are stronger than with MAPK, which may further suggest that Raf is the important component in driving the stabilisation of AP-1. This evidence suggests that two pathways to circumvent the need for hormones may be present and active in CRPC, and these allow tumours to continue to grow unabated without the need for hormones to drive proliferation and metastasis. Furthermore, it indicates that members of the Raf and MAPK pathways would be potential targets for intervention therapy in the treatment of CRPC tumours.

In addition, we have previously reported an increase in AR levels in 65% of CRPC cases (Edwards *et al*, 2001), which alone was not overly significant; however, combined with stabilisation through c-Jun and phosphorylation from the MAPK cascade, the small increase becomes highly significant with increased activity. This may also explain the correlation that exists between levels of MAPK in the nucleus and levels of AR found in CRPC tumours.

From the results presented here, it is evident that increasing levels of Raf-1 and/or MAPK are indicative of more rapid biochemical relapse and a more rapid decline into CRPC, which ultimately results in reduced survival times, with tumours showing more severe and aggressive action. This suggests that these members of this pathway are vital components and may be used as indicators of disease severity, providing a more accurate system for grading prostate cancer, in particular CRPC, than other systems in use, for example ,breast cancer (Galea *et al*, 1992), which have proven insufficient in classifying prostate cancer. Furthermore, it suggests that these pathways are involved in the development of CRPC. Ultimately, the Raf and MAPK pathways may turn out to be excellent targets for preventative therapy, as well as providing potential prognostic and/or diagnostic markers.

## Figures and Tables

**Table 1:** Protein histoscores for phosphorylated Raf in HNPC and CRPC tumours

	**HNPC**	**CRPC**	***P*-value**	**% Fall**	**% Unchanged**	**% Rise**
pRaf (Ser259)	127.5 (105–160)	137.5 (89–175)	0.42	14 (9/65)	69 (45/65)	17 (11/65)
pRaf (Ser338) nuclear	55 (0–125)	50 (0–200)	0.22	16 (8/50)	54 (27/50)	30 (15/50)
pRaf (Ser338) cytoplasmic	100 (20–114)	92.5 (23–103)	0.43	32 (16/50)	40 (20/50)	28 (14/50)

Abbreviations: CRPC=castrate-resistant prostate cancer; HNPC=hormone-naïve prostate cancer.

**Table 2:** MAPK protein histoscores in HNPC and CRPC tumours

	**HNPC**	**CRPC**	***P*-value**	**% Fall**	**% Unchanged**	**% Rise**
MAPK (nuclear)	60 (20–100)	80 (30–100)	0.27	27 (15/56)	39 (22/56)	34 (19/56)
MAPK (cytoplasm)	150 (120–190)	160 (130–190)	0.52	18 (10/56)	59 (33/56)	23 (13/56)
pMAPK (nuclear)	90 (32–144)	90 (52–119)	0.82	23 (13/56)	50 (28/56)	27 (15/56)
pMAPK (cytoplasm)	120 (76–160)	120 (92–150)	0.93	20 (11/56)	60 (34/56)	20 (11/56)

Abbreviations: CRPC=castrate-resistant prostate cancer; HNPC=hormone-naïve prostate cancer; MAPK=mitogen-activated protein kinase.
