# Supplementary material for: Upregulation of MAPK pathway is associated with survival in castrate-resistant prostate cancer
Source: Br J Cancer. 2011 May 10;104(12):1920–8. doi: 10.1038/bjc.2011.163 (PMC3111196; doi:10.1038/bjc.2011.163)
Supplement: Supplementary Figure 1 [file bjc2011163x1.doc]

**Supplementary Figure 1.**

A)

B)

C)

D)

**Supplementary Figure 1. PSA and Gleason Score Relapse (HNPC) and Survival (CRPC) Rates in Prostate Cancer. A) Time to relapse is independent of Gleason Score; B) Time to death is not related to Gleason Score; C) Time to relapse is not dependent on PSA level, shown in quartile ranges; D) Time to death is not related to PSA level, shown in quartile ranges.**
